# FedAAR: A Novel Federated Learning Framework for Animal Activity Recognition with Wearable Sensors

**DOI:** 10.3390/ani12162142

**Published:** 2022-08-21

**Authors:** Axiu Mao, Endai Huang, Haiming Gan, Kai Liu

**Affiliations:** 1 Department of Infectious Diseases and Public Health, Jockey Club College of Veterinary Medicine and Life Sciences, City University of Hong Kong, Hong Kong 999077, China; 2 Department of Computer Science, City University of Hong Kong, Hong Kong 999077, China

**Keywords:** data privacy, animal behaviour, deep learning, distributed learning, client-drift, local gradient conflicts

## Abstract

**Simple Summary:**

Automated animal activity recognition has achieved great success due to the recent advances in deep learning, allowing staff to identify variations in the animal behavioural repertoire in real-time. The high performance of deep learning largely relies on the availability of big data, which inevitably brings data privacy issues when collecting a centralised dataset from different farms. Federated learning provides a promising solution to train a shared model by coordinating multiple farms (clients) without sharing their private data, in which a global server periodically aggregates local (client) gradients to update the global model. The study develops a novel federated learning framework called FedAAR to achieve automated animal activity recognition using decentralised sensor data and to address, in particular, two major challenges resulting from data heterogeneity when applying federated learning in this task. The experiments demonstrate the performance advantages of FedAAR compared to the state-of-the-art, proving the promising capability of our framework for enhancing animal activity recognition performance. This research opens new opportunities for developing animal monitoring systems using decentralised data from multiple farms without privacy leakage.

**Abstract:**

Deep learning dominates automated animal activity recognition (AAR) tasks due to high performance on large-scale datasets. However, constructing centralised data across diverse farms raises data privacy issues. Federated learning (FL) provides a distributed learning solution to train a shared model by coordinating multiple farms (clients) without sharing their private data, whereas directly applying FL to AAR tasks often faces two challenges: client-drift during local training and local gradient conflicts during global aggregation. In this study, we develop a novel FL framework called FedAAR to achieve AAR with wearable sensors. Specifically, we devise a prototype-guided local update module to alleviate the client-drift issue, which introduces a global prototype as shared knowledge to force clients to learn consistent features. To reduce gradient conflicts between clients, we design a gradient-refinement-based aggregation module to eliminate conflicting components between local gradients during global aggregation, thereby improving agreement between clients. Experiments are conducted on a public dataset to verify FedAAR’s effectiveness, which consists of 87,621 two-second accelerometer and gyroscope data. The results demonstrate that FedAAR outperforms the state-of-the-art, on precision (75.23%), recall (75.17%), F1-score (74.70%), and accuracy (88.88%), respectively. The ablation experiments show FedAAR’s robustness against various factors (i.e., data sizes, communication frequency, and client numbers).

## 1. Introduction

Monitoring and assessing animal activities provide rich insights into their physical status and circumstances, as activity is one of the most critical indicators of animal health and welfare [1]. Traditionally, animal activity monitoring largely relies on direct visual and behavioural observation, which is time-consuming and labour-intensive [2]. Over the past decade, automated animal activity recognition (AAR) with wearable sensors, which allows staff to identify variations in the animal behavioural repertoire in real-time, has attracted increasing attention and achieved great success. In the sensor-based AAR systems, wearable sensors are attached to a certain part of the animal body (e.g., ear, neck, halter, back, and leg) to collect motion data (e.g., acceleration and angular velocity), which are then used for classifying animal activities (e.g., feeding, drinking, and resting) with suitable classification algorithms.

In recent years, deep learning has dominated the tasks in AAR due to the high performance achievable with the help of large-scale training datasets [3,4]. For instance, convolutional neural networks (CNNs) are widely used to automatically classify various animal activities, such as the walking and ruminating of cattle [4], the trotting and cantering of equines [2], and the eating and petting of canines [5]. However, collecting a large corpus of centralised datasets from different sources (e.g., farms) often raises data privacy issues. Federated learning (FL) has recently emerged as a distributed learning paradigm, providing an attractive solution to this problem [6,7,8]. A standard FL system iterates two steps periodically, i.e., local training in farms (clients) and global aggregation in a trustworthy centre (server), to train a global model. Specifically, during local training, each client downloads the parameters of the global model from the server-side to initialise its local model and then exploits local data to calculate local (client) gradients, which are sent to the server in turn. The server collects these local gradients and aggregates them to update the global model. Such a training mechanism enables data owners to build a shared model collaboratively without exchanging their private data, effectively promoting privacy preservation between clients [9,10,11].

Despite remarkable benefits provided by FL, directly applying FL to AAR tasks often faces two major challenges: client-drift during local training and local gradient conflicts during global aggregation, which easily increase the difficulty in model convergence and cause extreme degradation of performance [12]. First, the movement patterns of individual animals are often drawn from distinct distributions, which inevitably results in data heterogeneity between clients. Such data heterogeneity enlarges the inconsistency of learned features across clients, easily raising drift concerns between client updates since each client model is optimised towards its local objective instead of global optima during local training [13,14,15]. To address this issue, some existing methods [14,15,16,17] impose constraints on the local optimisation by exploiting a model-level regularisation term, which aims to facilitate all local models to approach consistent views. For instance, FedProx restricted local model parameters to be close to global parameters by adding a proximal term in the local training [16]. SCAFFOLD corrected for client-drift by using control variates to overcome gradient dissimilarity in local training [15]. Both FedLSD and FedCAD regarded the distributed global model as a teacher and distilled its predictions on local data to guide local optimisation [13,17]. However, these methods only emphasised constraints on local models instead of directly forcing clients to learn consistent features, consequently yielding sub-optimal performance.

Second, gradients among clients often possess inconsistent directions and even have conflicting components due to the inconsistency between local optimisation objectives in the context of data heterogeneity. Directly aggregating all local gradients in standard FL methods easily leads to mutual interferences among clients’ knowledge, further hampering the process of model convergence and exacerbating the risk of model divergence. To alleviate this problem, most existing works [18,19,20,21] attempted to modify the global aggregation mechanism by dynamically adjusting aggregation weights to local gradients under different criteria. For example, IDA assigned each client weight based on the inverse distance of its gradient to the averaged gradient across all clients [21]. Precision-weighted FL aggregated local gradients by averaging gradients by the inverse of their estimated variance [19]. ABAVG combined gradients over clients by the accuracies of local models on the validation set at the server-side [18]. However, aggregating local gradients merely by changing their weights still cannot drastically remove conflicting components in gradients and may even lose important information in gradients.

In this study, we propose a novel FL framework, namely FedAAR, to achieve automated AAR by uniting decentralised data while tackling the abovementioned issues. To alleviate the client-drift issue, we design a prototype-guided local update (PLU) module, in which we introduce a globally shared prototype (classwise feature representation) as shared knowledge to constrain local optimisation. To reduce conflicts between local gradients, we devise a new gradient-refinement-based aggregation (GRA) module to constantly recalibrate local gradients throughout the training process. The proposed FedAAR is trained based on a public dataset [22], and its generalisation performance is compared with that of the state-of-the-art FL strategies and with the centralised learning algorithm. In summary, the main contributions of this study are as follows:

- We propose a novel FL framework called FedAAR to automatically recognise animal activities based on distributed data. This gives it the considerable potential that multiple farms jointly train a shared model using their private dataset while protecting data privacy and ownership. To the best of our knowledge, this is the first time the FL method has been explored in automated AAR across multiple data sources.
- To alleviate client-drift issues, we devise a PLU module to replace the traditional local training process. The PLU module forces all clients to learn consistent feature knowledge by imposing a global prototype guidance constraint to local optimisation, further reducing the divergence between client updates.
- Different from existing aggregation mechanisms, we design a GRA module to reduce conflicts among local gradients. The GRA module eliminates conflicting components between local gradients during global aggregation, effectively guaranteeing that all refined local gradients point in a positive direction to improve the agreement among clients.
- Our experimental results demonstrate that our proposed FedAAR outperforms the state-of-the-art FL strategies and exhibits performance close to that of the centralised learning algorithm. This proves the promising capability of our method to enhance AAR performance without privacy leakage. We also validate the performance advantages of our approach compared to the baseline in different practical scenarios (i.e., local data sizes, communication frequency, and client numbers), providing rich insights into the appropriate future applications of our method.

## 2. Materials and Methods

### 2.1. Data Description

The dataset used in this study is a public dataset created by [22]. This dataset is a centralised dataset comprising 87,621 two-second samples that were collected from six horses with neck-attached IMUs. The sampling rate was set to 100 Hz for both the tri-axial accelerometer and gyroscope and 12 Hz for the tri-axial magnetometer. Table 1 illustrates the data distribution of the dataset. Six activities (i.e., eating, galloping, standing, trotting, walking-natural, and walking-rider) were registered, and the number of activity samples of the six individuals (i.e., Happy, Zafir, Driekus, Galoway, Patron, and Bacardi) was 23,625, 11,071, 10,127, 24,602, 12,849, and 5347, respectively. As in our previous study set [23], we exploited the tri-axial motion data from the accelerometer and gyroscope as our input samples, which were normalised before being input into the network.

### 2.2. Preliminaries for Federated Learning

A federated learning (FL) system coordinates *K* clients to collectively train a global model while keeping their data stored locally, effectively reducing the potential for violating data privacy. In each client *k*, the local dataset ${\left\{\left(x_{i}^{k},\;y_{i}^{k}\right)\right\}}_{i=1}^{N^{k}}$ is sampled from a distribution *D^k^*, where $y_{i}^{k}\;\in$ {1,⋯, C} corresponds to the ground-truth label of the data instance $x_{i}^{k}$, *C* is the number of label categories, and *N^k^* is the data number of the *k*-th client. The training of the FL system mainly consists of *T* communication rounds between a global server and *K* clients, with the detailed procedure of each communication round divided into the following three steps:

**Step 1.**  All clients synchronously download a global model *w^global^* from a global server.
**Step 2.**  Each client *k* uses the global model *w^global^* to initialise its local model *w^k^* (i.e., $w_{initial}^{k}=w^{global}$) and then conducts local training for *E* epochs, i.e., minimising the local optimisation objective by using a gradient descent algorithm:

(1) $$\min\;\frac{1}{N^{k}}\sum_{i=1}^{N^{k}}{ℒ}_{CE}^{k}\left(w^{k},\;x_{i}^{k},\;y_{i}^{k}\right),$$

where ${ℒ}_{CE}^{k}$ denotes the cross-entropy loss function of the *k*-th client. After local training, we can obtain the updated local model $w_{updated}^{k}$ and local gradient *g^k^* (i.e., the difference between the updated local model $w_{updated}^{k}$ and the initial local model $w_{initial}^{k}$).

**Step 3.**  Each client *k* then uploads its local gradient *g^k^* to the global server. These local gradients ${\left\{g^{k}\right\}}_{k=1}^{K}$ from all clients are aggregated by directly averaging to generate global gradients *g^global^*:

(2) $$g^{global}=\frac{1}{K}\overset{K}{\underset{k=1}{\sum }}g^{k}.\;$$

Afterwards, the global gradient *g^global^* is used to further update the original global model *w^global^*:

(3) $$w^{global}=w^{global}+g^{global}.$$

The updated global model *w^global^* will be sent to all clients again in the next communication round (**Step 1**). The above steps are performed repeatedly until the global model achieves convergence.

The implementation of standard FL (e.g., FedAvg) heavily relies on the assumption that no data heterogeneity issues occur across clients (i.e., data between clients follow a uniform distribution). However, this assumption does not hold in AAR tasks because the discrepancy of movement patterns among individual animals often results in data heterogeneity, thus giving rise to detrimental effects on the training of FL. Specifically, data heterogeneity across clients inevitably enlarges the inconsistency of learned features between clients, thereby inducing drift between client updates as each client model is optimised toward its local objective instead of global optima [17]. In addition, local gradients may conflict with each other due to the inconsistent local objectives, resulting in knowledge interference among clients when directly aggregating all local gradients. Hence, there is a need to reformulate the above optimisation process to adapt to AAR tasks.

### 2.3. Our Proposed FL Framework for AAR

#### 2.3.1. Overview

**Figure 1:**
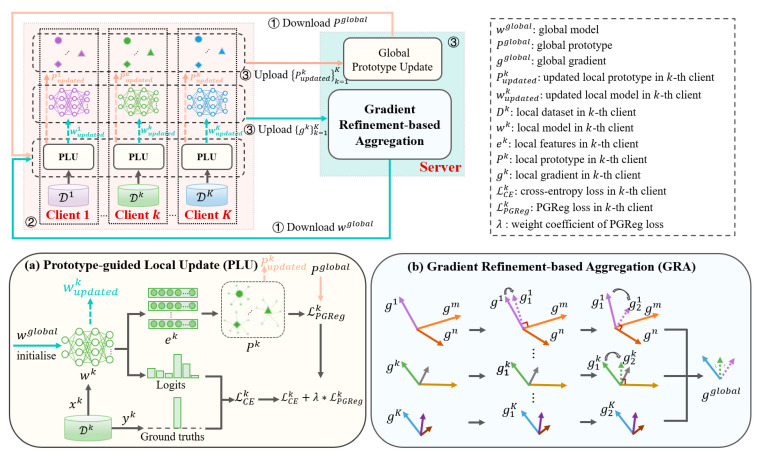
Overall architecture of our proposed FedAAR framework. The training of FedAAR consists of *T* communication rounds between a global server and *K* clients, where the workflow of each communication round includes three steps as follows. ① Each client *k* first downloads the same global model *w^global^* and global prototype $P^{global}$ from a global server simultaneously. ② Based on the local dataset *D^k^* in each client *k*, the local model *w^k^* is initialised and then trained in a prototype-guided local update (PLU) module (Figure 1**a**). After local training, the updated local model $w_{updated}^{k}$ and local prototype $P_{updated}^{k}$ can be obtained. The local gradient *g^k^* can be calculated as the difference between the updated local model $w_{updated}^{k}$ and the initial local model (i.e., the downloaded global model *w^global^*). ③ All local gradients ${\left\{g^{k}\right\}}_{k=1}^{K}$ and local prototypes ${\left\{P_{updated}^{k}\right\}}_{k=1}^{K}$ are then uploaded to the global server. Afterwards, these local gradients ${\left\{g^{k}\right\}}_{k=1}^{K}$ are aggregated to a global gradient $g^{global}$ using a gradient-refinement-based aggregation (GRA) module (Figure 1**b**) and then used to update the global model *w^global^*. In addition, local prototypes ${\left\{P_{updated}^{k}\right\}}_{k=1}^{K}$ are aggregated to update the global prototype $P^{global}$. The updated global model *w^global^* and global prototype $P^{global}$ are sent to all clients again in the next communication round. The above three processes are repeated until the global model achieves convergence.

In this study, we propose a novel FL framework called FedAAR to achieve automated AAR in the context of data heterogeneity between clients, as illustrated in Figure 1. The training of FedAAR consists of *T* communication rounds between a global server and *K* clients, where the detailed procedures of each communication round include three steps as follows. (1) Each client *k* first downloads the same global model *w^global^* and global prototype $P^{global}$ from a global server simultaneously. (2) Based on the local dataset $D^{k}$ in each client *k*, the local model $w^{k}$ is initialised as the global model *w^global^* and then trained in a prototype-guided local update (PLU) module (Figure 1a; see Section 2.3.2). After local training, the updated local model $w_{updated}^{k}$ and local prototype $P_{updated}^{k}$ can be obtained. Then, the local gradient $g^{k}$ can be calculated as the difference between the updated local model $w_{updated}^{k}$ and the initial local model (i.e., the downloaded global model *w^global^*). (3) All local gradients ${\left\{g^{k}\right\}}_{k=1}^{K}$ and local prototypes ${\left\{P_{updated}^{k}\right\}}_{k=1}^{K}$ are then uploaded to the global server simultaneously. Afterwards, these local gradients ${\left\{g^{k}\right\}}_{k=1}^{K}$ are aggregated to a global gradient $g^{global}$ using a gradient-refinement-based aggregation (GRA) module (Figure 1b; see Section 2.3.3) and then used to update the global model *w^global^* (see Equation (3)). In addition, local prototypes ${\left\{P_{updated}^{k}\right\}}_{k=1}^{K}$ are aggregated to update the global prototype $P^{global}$. The updated global model *w^global^* and global prototype $P^{global}$ are sent to all clients again in the next communication round. The above three processes are repeated until the global model achieves convergence.

#### 2.3.2. Prototype-Guided Local Update

To alleviate client-drift issues in the local training, we devised a PLU module (Figure 1a), which introduces a global prototype to serve as the shared feature knowledge to guide local optimization.

First, each client *k* simultaneously downloads the same global model *w^global^* and global prototype $P^{global}$ from the server. Based on the local dataset *D^k^* in each client *k*, the local model *w^k^* is initialised as the global model *w^global^* and then trained in the proposed PLU module. Specifically, given batchwise samples ${\left\{\left(x_{i}^{k},\;y_{i}^{k}\right)\right\}}_{i=1}^{ℬ}$ with *C* categories in client *k*, we first adopted the feature extractor to extract feature representation ${\left\{e_{c,i}^{k}\right\}}_{i=1}^{{ℬ}_{c}}$ for samples in each class *c*, where *B* and *B_c_* represent the number of total samples and the c-th categorial samples in a batch, respectively. These features are then used to calculate the corresponding classwise prototype ${\left\{P_{c}^{k}\right\}}_{c=1}^{C}$, where $P_{c}^{k}$ is the mean value of feature representations of samples in class *c*, i.e.,

(4) $$P_{c}^{k}=\overset{{ℬ}_{c}}{\underset{i=1}{\sum }}\frac{e_{c,i}^{k}}{{ℬ}_{c}}.$$

Herein, we empirically used the network that removed the last fully connected layer as the feature extractor [24].

Inspired by prototype learning, in which gathering the prototypes across heterogeneous datasets enables the incorporation of feature representations over various data distributions [24,25], we brought a global prototype ${\left\{P_{c}^{global}\right\}}_{c=1}^{C}$ ($P^{global}$) aggregated across clients as consistent feature-level views to guide local training. We propose a new prototype guidance regularisation (PGReg) loss ${ℒ}_{PGReg}^{k}$ as follows:

(5) $${ℒ}_{PGReg}^{k}=\sum_{c\in C}{||P_{c}^{k}-P_{c}^{global}||}_{2},$$

where $P_{c}^{global}$ denotes the global prototype of the c-th category and ${||\cdot ||}_{2}$ denotes the L2 distance. The PGReg loss sufficiently encourages local prototype ${\left\{P_{c}^{k}\right\}}_{c=1}^{C}$ of each client to approach the same global prototype ${\left\{P_{c}^{global}\right\}}_{c=1}^{C}$, effectively keeping all clients as having a consistent direction of feature learning. Thus, the total loss function can be reformulated as the linear combination of the original classification loss ${ℒ}_{CE}^{k}$ and PGReg loss ${ℒ}_{PGReg}^{k}$:

(6) $${ℒ}^{k}={ℒ}_{CE}^{k}+\lambda *{ℒ}_{PGReg}^{k},$$

where *λ* is the weight coefficient of PGReg loss and its value equals 0 in the initial state. Then, we conducted the local training (see Equation (1)) to obtain the updated local model $w_{updated}^{k}$. The updated local prototype ${\left\{P_{c,updated}^{k}\right\}}_{c=1}^{C}$ can be computed based on the feature vectors of correctly classified samples using the updated local model $w_{updated}^{k}$. The local gradient $g^{k}$ can be calculated as the difference between the updated local model $w_{updated}^{k}$ and the initial local model (i.e., the downloaded global model $w^{global}$). Afterwards, all local gradients ${\left\{g^{k}\right\}}_{k=1}^{K}$ and local prototypes ${\left\{P_{updated}^{k}\right\}}_{k=1}^{K}$ are uploaded to the server to update the original global model and global prototype.

At the server-side, to avoid the attack resulting from noise components involved in the updated local prototypes ${\left\{P_{updated}^{k}\right\}}_{k=1}^{K}$, we devised a novel adaptive global prototype update process. Instead of directly replacing the global prototype with the average values of local prototypes over clients, we define the updated global prototype ${\left\{P_{c}^{global}\right\}}_{c=1}^{C}$ ($P^{global}$) as a linear combination of the weighted averaged local prototypes ${\left\{{\bar{P}}_{c}\right\}}_{c=1}^{C}$ and the original global prototype ${\left\{P_{c}^{global}\right\}}_{c=1}^{C}$:

(7) $$P_{c}^{global}=\gamma_{c}*P_{c}^{global}+\left(1-\gamma_{c}\right)*{\bar{P}}_{c},$$

where *γ_c_* is an adaptively balanced coefficient controlling the updating degree of the global prototype and ${\bar{P}}_{c}=\overset{K}{\underset{k=1}{\sum }}n_{c}^{k}P_{c,updated}^{k}/\overset{K}{\underset{k=1}{\sum }}n_{c}^{k}$, where $n_{c}^{k}$ represents the number of correctly classified samples belonging to the c-th category in client *k*. Considering that the updated global prototype may be transferred close to each other due to noise components, inducing similarity increases of inter-class feature vectors [26], we modulated *γ_c_* according to the intra-class and inter-class distance between local prototypes and the original global prototype:

(8) $$\gamma_{c}=\frac{exp^{d\left({\bar{P}}_{c},\;P_{c}^{global}\right)}}{exp^{d\left({\bar{P}}_{c},\;P_{c}^{global}\right)}+exp^{d\left({\bar{P}}_{c},\;P_{c^{\prime }}^{global}\right)}},$$

where d(·) denotes the Euclidean distance and $P_{c^{\prime }}^{global}$ is the global prototype of class *c*’, that is the closest class to class *c*, i.e., $c^{\prime }=\underset{j\in \left\{1,2,\ldots ,C\right\}\backslash c}{\mathrm{argmin}}d\left(P_{c}^{global},\;P_{j}^{global}\right)$. Intuitively, when the averaged local prototype ${\bar{P}}_{c}$ is farther from the global prototype of the same class *c* than the global prototype of its closest class (i.e., $d\left({\bar{P}}_{c},\;P_{c}^{global}\right)>d\left({\bar{P}}_{c},\;P_{c^{\prime }}^{global}\right)$), the contribution of ${\bar{P}}_{c}$ on the update process (Equation (7)) should be lower than that of $P_{c}^{global}$. Note that when the value of $P_{c}^{global}$ is empty at the early training phase, we directly put the updated averaged prototype ${\bar{P}}_{c}$ into global prototype $P_{c}^{global}$.

#### 2.3.3. Gradient-Refinement-Based Aggregation

To reduce conflicts among local gradients during global aggregation, we designed a new GRA module (Figure 1b), which eliminates the conflicting components between local gradients, ensuring all refined local gradients point in a positive direction to improve the agreement across clients.

**Figure 2:**
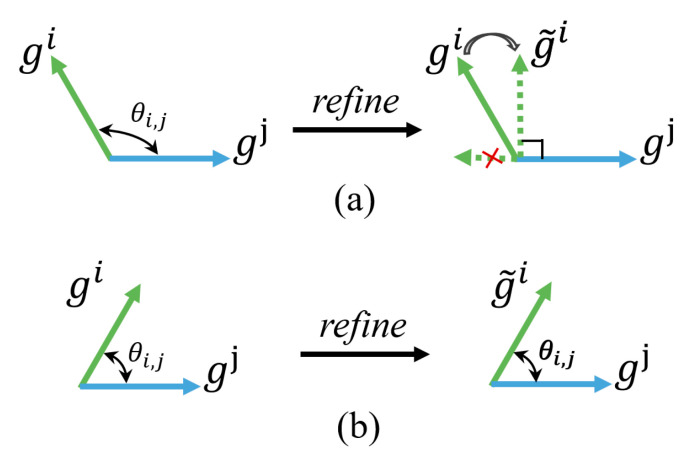
Process of gradient refinement. Here, $\theta_{i,j}$ represents the angle between two local gradients $g^{i}$ and $g^{j}$ and ${\tilde{g}}^{i}$ represents the refined local gradients in client i. (**a**) When gradients $g^{i}$ and $g^{j}$ are conflicting (i.e., $\cos\theta_{i,j}$ < 0), the gradient $g^{i}$ is replaced with its projection ${\tilde{g}}^{i}$ onto the normal plane of $g^{j}$. (**b**) When gradients $g^{i}$ and $g^{j}$ are non-conflicting (i.e., $\cos\theta_{i,j}$ > 0), gradient $g^{i}$ is unchanged (i.e., ${\tilde{g}}^{i}=g^{i}$).

Given a set of local gradients ${\left\{g^{k}\right\}}_{k=1}^{K}$ that are uploaded to the global server, we first characterised any two of these gradients as conflicting when their directions point away from one another (i.e., having a negative cosine similarity). Herein, we aimed to reconstruct consensus vectors by refining the conflicting local gradients and keeping the non-conflicting local gradients invariant. Figure 2 visualises the main step of the local gradient refinement process. Specifically, suppose *g^i^* is the local gradient at the *i*-th client and *g^j^* is selected in a random order from the rest of the local gradients, where i∈{1,2,…,K} and j∈{1,2,…,i−1,i+1,…,K}. The cosine similarity between *g^i^* and *g^j^* can be denoted as $\cos\theta_{i,j}$, where $\theta_{i,j}$ is the angle between *g^i^* and *g^j^*. As shown in Figure 2a, if *g^i^* conflicts with *g^j^* (i.e., cosine similarity $\cos\;\theta_{i,j\;}$< 0), we remove the component of *g^i^* in the direction fully opposite that of $g^{j}$ and alter $g^{i}$ by its projection ${\tilde{g}}^{i}$ onto the normal plane of *g^j^*:

(9) $${\tilde{g}}^{i}=g^{i}-\frac{g^{i}\cdot g^{j}}{||g^{j}||{}^{2}}g^{j}.\;$$

If *g^i^* and *g^j^* are not in conflict (i.e., cosine similarity $\cos\theta_{i,j}\;$ > 0), we retain the original local gradient *g^i^* as unchanged (i.e., ${\tilde{g}}^{i}=g^{i}$), as shown in Figure 2b. Afterward, the updated ${\tilde{g}}^{i}$ is further selectively updated according to the condition of whether there are conflicting components compared to other local gradients. This process is repeated until all of the local gradients are compared.

Supposing ${\left\{{\tilde{g}}^{k}\right\}}_{k=1}^{K}$ is a collection of refined local gradients, we then aggregate them using Equations (2) and (3) to further update the global model $w^{global}$.

### 2.4. Evaluation Methods

The precision, recall, F1-score, and accuracy were measured to indicate the comprehensive performance of the classification model. They are defined as follows:

(10) $$Precision=\frac{TP}{TP+FP}\times 100\%,\;$$

(11) $$Recall=\frac{TP}{TP+FN}\times 100\%,$$

(12) $$F1-score=\frac{2TP}{2TP+FP+FN}\times 100\%,$$

(13) $$Accuracy=\frac{TP+TN}{TP+TN+FP+FN}\times 100\%,$$

where *TP*, *FP*, *TN*, and *FN* are the number of true positives, false positives, true negatives, and false negatives, respectively.

### 2.5. Implementation Details

In our previous work, we established a cross-modality interaction network (CMI-Net) for equine activity recognition based on accelerometer and gyroscope data [23]. The CMI-Net consists of a dual CNN trunk architecture and a joint cross-modality interaction module, effectively improving the classification performance for equine activities. Herein, we used CMI-Net as the global classification model to achieve our proposed FedAAR.

During training, we used softmax cross-entropy loss with L2 regularisation (a weight decay of 0.15). An Adam optimiser with an initial learning rate of 5 × 10^−5^ was used, and the learning rate decreased by 0.1 times every 20 epochs. The communication rounds *T* and batch size were set to 100 and 256, respectively. If not specified, the value of local training epochs *E* was set to 1, and the weighting coefficient *λ* in PLU was set to 0.05 by default. The global model was initialised randomly before being downloaded to clients in the first round. Over the communication rounds, the best global model with the highest testing accuracy was saved as the optimal model. To verify the model’s generalisation abilities, we performed the leave-one-out-based validation method. Specifically, we separately ran three times in each experiment. In each run (time), we randomly selected five horses from the original six horses and individually assigned these five horses to five farms (clients). Each of these five horses’ data serves as each client’s data, and all client data were used as training data to train a shared global model collectively. The data from the remaining horse (the sixth horse) were then used as the test data to verify the performance of the trained global model. The final test result of the model performance is presented in the format mean ± std from the three runs. This kind of data allocation can well simulate practical scenarios, i.e., data heterogeneity across farms, since the movement patterns of individual animals are often drawn from distinct distributions. All experiments were executed using the PyTorch framework on an NVIDIA Tesla V100 GPU. The source code is available at https://github.com/Max-1234-hub/FedAAR (accessed on 20 June 2022).

## 3. Results and Discussion

Overall, the experimental results demonstrate that our proposed FedAAR outperforms the state-of-the-art FL strategies from both quantitative and qualitative perspectives while exhibiting performance close to that of the centralised learning algorithm. Ablation studies were then carried out to evaluate the effectiveness of the PLU and GRA module on the classification capability. In addition, a comprehensive investigation of FedAAR’s performance was conducted at different levels of three practical conditions (i.e., dataset sizes on local clients, communication frequency between local clients and the global server, and client numbers). The experimental results of FedAAR were compared with those of its corresponding baseline, further validating the performance advantages of our method. In the end, some possible future research directions are proposed. The details are described as follows.

### 3.1. Comparisons with State-of-the-Art Methods

*Quantitative comparison:* We compared the performance of our proposed FedAAR with the state-of-the-art FL approaches (i.e., FedAvg, FedPorx, IDA, SiloBN, FedBN, and precision-weighted FL) and with centralised learning. As illustrated in Table 2, our proposed framework outperformed all of the selected state-of-the-art FL methods, with the highest average values of 75.23%, 75.17%, 74.70%, and 88.88% in precision, recall, F1-score, and accuracy, respectively. These results demonstrate the promising capabilities of FedAAR for animal behavioural classification. In particular, compared with the precision-weighted FL [19], which obtains relatively good performance among the selected state-of-the-art FL methods, our proposed approach achieves remarkable increments of 3.75%, 9.39%, 8.34%, and 4.22% in the average values of the precision, recall, F1-score, and accuracy, respectively. This can be ascribed to the ability of our architecture to effectively alleviate client-drift concerns in local training and conflicts of local gradients during global aggregation. Compared with centralised learning, which provides the upper bounds, the performance of our method is close, with 3.64%, 3.26%, and 3.49% lower average values of the recall, F1-score, and accuracy, respectively. This further reveals the favourable performance of our method. It is also worth noting that the proposed approach demonstrates smaller variances than the state-of-the-art works, with 1.01%, 3.92%, 2.49%, and 1.36% variance in the precision, recall, F1-score, and accuracy, respectively, indicating the good stability and robustness of our approach.

**Figure 3:**
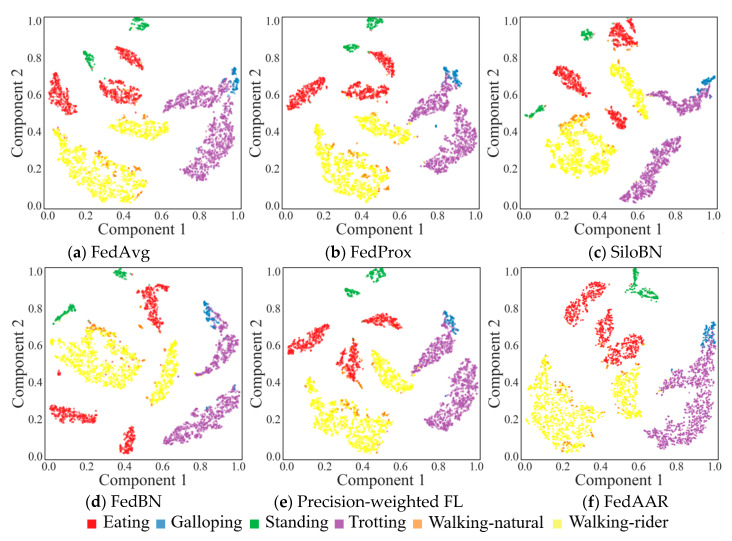
t-SNE visualisation of the feature vectors produced by our proposed FedAAR and other FL approaches.

*Qualitative comparison:* To qualitatively verify our proposed approach, we visualise the feature vectors of the test set before the last fully connected layer within FedAAR and the state-of-the-art FL models, with the help of t-distributed stochastic neighbour embedding (t-SNE) [29]. As illustrated in Figure 3, the two-dimensional embeddings can reflect the distribution of the network features in the feature space and indicate the generalisation ability of models, in which each point corresponds to a sample and different colours represent different category labels (ground-truth). Better generalisation means that the feature points of samples belonging to the same class cluster closer to each other, whereas the points between different classes are located far from each other. From the embedding visualisation, we can observe that the proposed FedAAR displays more compact clusters within the same categories and larger distances between different categories compared to the selected state-of-the-art methods. This reflects the success of our approach in improving the consistency of update directions across clients from both the local optimisation and global aggregation perspectives, which is beneficial to the generalisation performance promotion of the global model.

### 3.2. Ablation Studies

#### 3.2.1. Evaluation of PLU and GRA Module

*Quantitative analysis:* To investigate the effectiveness of PLU and GRA on classification performance, we designed four different experimental settings as follows. (1) Baseline: we applied FedAvg as our baseline for the ablative comparison; (2) Baseline + PLU: we used the PLU module instead of the original optimisation process in the baseline during local training; (3) Baseline + GRA: we replaced the original weighted average mechanism in the baseline with our proposed GRA module during global aggregation; (4) FedAAR: we used our proposed framework involving both PLU and GRA modules simultaneously. The quantitative results are shown in Table 3. It is remarkable that the two modules individually yield desirable performance improvements over the baseline, which proves that each of the PLU and GRA modules plays an important role in AAR tasks with data heterogeneity issues. In particular, the GRA module contributes to the tremendous performance improvements, with increases of 3.07%, 9.23%, 8.34%, and 3.47% in the average values of the precision, recall, F1-score, and accuracy, respectively. The variances also significantly decline, by 3.47%, 6.85%, 7.63%, and 2.27% in the precision, recall, F1-score, and accuracy, respectively, when the GRA module is used separately. These experimental results validate the significance of the GRA module in the model’s performance and robustness improvements. The inclusion of the PLU module in addition to the GRA module enables all clients to possess consistent guidance directions of feature learning and further obtain relative gains of 1.06%, 0.98%, 0.96%, and 1.10% in the average values of the precision, recall, F1-score, and accuracy, respectively.

*Analysis of the hyper-parameter*λ*in PLU:* The hyper-parameter λ in Equation (6) represents the weight of newly added PGReg loss, corresponding to the constraint degree of global knowledge on local training. We conducted experiments to evaluate the performance of our approach with different λ values (i.e., 0.01, 0.03, 0.05, 0.07, and 0.09), with the results shown in Table 4. The FedAAR achieves clearly the best average values in the recall and F1-score when λ was set to 0.05, while obtaining a performance in the precision and accuracy comparable to the model with λ set to 0.09. Although the precision and accuracy arrive at the highest average values when λ was set to 0.09, the model exhibits a poor recall and F1-score. In addition, the average recall and F1-score values first increase and then decrease as λ varied from 0.01 to 0.09, which illuminates the likely benefit of properly choosing the value of λ for the improvement of overall classification performance.

**Figure 4:**
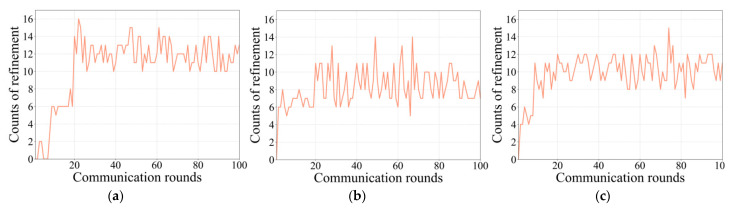
Counts of refinement operations during the training process over three runs from (**a**–**c**).

*Visualisation of refinements in GRA:* To provide further insights into the enormous contributions of the GRA module, we visualise the counts of gradient refinement operations during the training process over three runs in Figure 4. It can be observed that various numbers of gradient modulation operations occur in each communication round, which confirms that conflicts among local gradients arise continuously during the training process. In addition, this observation implies that our framework can constantly and steadily recalibrate the local gradients across clients, effectively enhancing the model’s performance. This finding also reinforces the suitability of the proposed GRA module for AAR tasks in the context of data heterogeneity.

#### 3.2.2. Analysis of Local Dataset Size

**Figure 5:**
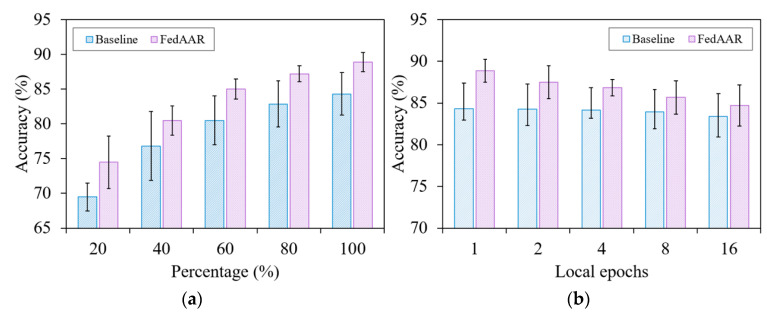
Testing accuracy of FedAAR and its baseline over varying (**a**) local dataset sizes and (**b**) local updating epochs.

To observe the behaviour of our proposed method over different data capacities, we present in Figure 5a the average testing accuracy of FedAAR and the baseline under various local dataset percentages (i.e., 20%, 40%, 60%, 80%, and 100%). The test accuracies of both FedAAR and the baseline decrease gradually as the number of training samples reduces, but the accuracy of FedAAR continues to exceed that of the baseline, validating the performance advantages of our method under scenarios with a small amount of data. In addition, FedAAR conducted on 60% local data still obtains higher accuracy than the baseline performed on full-sized local data, which reveals that our approach can effectively mitigate the performance degradation due to the reduced data amount.

#### 3.2.3. Analysis of Communication Frequency

The communication frequency between local clients and the global server may influence learning behaviour. We decreased the communication frequency by increasing the local updating epochs and present in Figure 5b the average testing accuracy of FedAAR and the baseline on various local updating epochs (i.e., *E* = 1, 2, 4, 8, 16). As expected [28], both FedAAR and the baseline achieve higher testing accuracy under smaller local updating epochs, because aggregating at lower frequencies (i.e., larger local updating epochs) easily results in the models’ divergence, especially in the early training stages [6]. Notably, FedAAR with a local epoch of 16 still exhibits higher accuracy than the baseline with one local epoch, demonstrating the superiority and reliability of our approach.

#### 3.2.4. Analysis of Client Numbers

**Figure 6:**
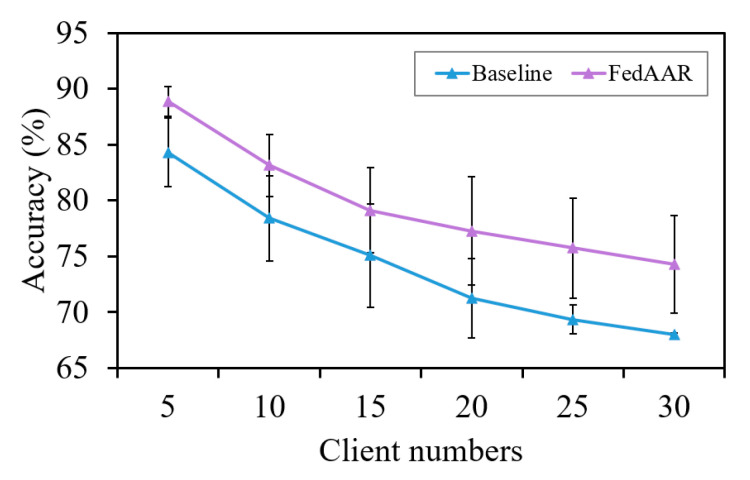
Testing accuracy of FedAAR and its baseline over various client numbers.

A larger number of clients may bring more conflicts among local gradients, which poses a great challenge to the practical application of FL. To further verify the performance advantages of FedAAR compared to the baseline under scenarios with more clients, we simulated the situations with varying client numbers by redistributing the original data based on the basic setting with five clients (see Section 2.5). Specifically, we separately parcelled each of five horse datasets into 2, 3, 4, 5, and 6 smaller ones, each serving as the training data of a single client, thus forming five settings with total client numbers of 10, 15, 20, 25, and 30, respectively. The data from the remaining horse were still used as the test data to validate our method’s performance. As shown in Figure 6, the testing accuracy consistently decreases as client numbers increase, but FedAAR drops more slowly than the baseline, indicating the robustness and scalability of our approach under scenarios with more clients.

### 3.3. Limitations and Implications

Due to its privacy-preserving nature, FL has been increasingly adapted to various applications, including mobile edge devices, industrial engineering, and health care [30,31,32]. As far as we know, no previous work has developed FL frameworks tailored for automated AAR applications based on decentralised data. Our method is the first to exploit FL to achieve animal behavioural recognition using distributed data without privacy leakage, opening up new opportunities for developing animal monitoring systems with strong robustness and generalisation capabilities.

Our method additionally requires the transmission of prototypes between clients and the server, increasing the dissemination of client information and communication overhead. However, this does not raise privacy concerns because prototypes only represent the average statistics across all already compressed local features [33]. In addition, the transmitted prototypes between the server and each client occupied 3 KB, which is only 2.35% of the communication cost of model parameters/gradients. This further reinforces the suitability of our proposed algorithm in practice.

Our method achieves automated AAR based on distributed data in the context of data heterogeneity. The major limitation is that our approach remains at the experimental level. To simulate practical scenarios, i.e., data heterogeneity across farms (clients), we assigned the horse dataset to multiple clients according to individuals since the movement patterns of individual animals are often drawn from distinct distributions. This kind of data allocation refers to some previous works [6,15,34], which generally divide a single centralised dataset into small ones. In addition, we separately selected one horse dataset (excluded in the training set) as the test set to test the trained model’s performance in each run of the experiment, which effectively promotes the generalisation capabilities of the model. In the future, we will collect more data from different farms and further verify the effectiveness of our proposed FL strategy.

Considering real-world applications, it is impossible for the client data to be fully labelled due to the time-consuming and costly process of data annotation. Thus, promising solutions are required for integrating FL with potential techniques (e.g., semi-supervised learning) for exploiting the unlabelled data sufficiently. In addition, our method can be extended to AAR tasks with other forms of data (e.g., visual) and different kinds of production management systems that need to use cross-silo data, further promoting the development of agriculture.

## 4. Conclusions

In this study, we developed a novel FL framework called FedAAR involving a PLU module and a GRA module to achieve automated AAR by uniting decentralised sensor data while avoiding privacy leakage. The PLU module forces all clients to learn consistent classwise feature representations in local training, effectively reducing drift among client updates. The GRA module eliminates the conflicting components between local gradients during global aggregation, which ensures that all refined local gradients point in a positive direction to improve the agreement among clients. The experimental results reveal that our approach outperforms the state-of-the-art FL methods and achieves performance that is close to that of the centralised learning algorithm. Ablation studies further illuminated the effectiveness of the PLU and GRA modules. In addition, comparative analyses of the performance of FedAAR and the baseline at different levels of three practical conditions (i.e., local data sizes, communication frequency, and client numbers) confirm the performance advantages of our algorithm. These analyses also provide rich insights into the appropriate future applications of our method.

## Figures and Tables

**Table 1:** Number of data samples per subject and activity.

	Activity	Eating	Galloping	Standing	Trotting	Walking-Natural	Walking-Rider	Total
Subject								
Happy		5063	696	1186	7038	746	8896	23,625
Zafir		1091	835	347	3559	161	5078	11,071
Driekus		2496	323	341	2673	270	4024	10,127
Galoway		4331	1043	1750	6423	1402	9653	24,602
Patron		1951	714	1244	3402	388	5150	12,849
Bacardi		1116	328	245	1981	360	1317	5347

**Table 2:** Comparative results (mean ± std) of our proposed FedAAR with state-of-the-art federated learning (FL) methods. The best two results for each metric are highlighted in bold.

Method	Precision (%)	Recall (%)	F1-Score (%)	Accuracy (%)
Centralised learning [23]	**83.34 ± 10.81**	**78.81 ± 2.40**	**77.96 ± 2.28**	**92.37 ± 3.84**
FedAvg [6]	71.10 ± 4.42	64.96 ± 9.81	65.40 ± 8.93	84.31 ± 3.05
FedProx [16]	71.10 ± 4.42	64.93 ± 9.83	65.37 ± 8.96	84.30 ± 3.06
IDA [21]	70.67 ± 5.45	64.35 ± 10.68	64.27 ± 10.02	84.36 ± 3.33
SiloBN [27]	71.15 ± 2.73	64.57 ± 9.41	64.96 ± 7.94	83.18 ± 2.64
FedBN [28]	70.90 ± 2.84	65.45 ± 8.81	65.82 ± 7.14	83.72 ± 2.16
Precision-weighted FL [19]	71.48 ± 3.78	65.78 ± 9.15	66.36 ± 8.03	84.66 ± 2.68
FedAAR (ours)	**75.23 ± 1.01**	**75.17 ± 3.92**	**74.70 ± 2.49**	**88.88 ± 1.36**

**Table 3:** Evaluation results (mean ± std) of the GRA and PLU modules on classification performance. The best results for each metric are highlighted in bold.

Method	Precision (%)	Recall (%)	F1-Score (%)	Accuracy (%)
Baseline	71.10 ± 4.42	64.96 ± 9.81	65.40 ± 8.93	84.31 ± 3.05
Baseline + PLU	73.04 ± 2.89	65.98 ± 9.34	67.10 ± 8.02	84.92 ± 3.00
Baseline + GRA	74.17 ± 0.95	74.19 ± 2.96	73.74 ± 1.30	87.78 ± 0.78
FedAAR	**75.23 ± 1.01**	**75.17 ± 3.92**	**74.70 ± 2.49**	**88.88 ± 1.36**

**Table 4:** Experimental results (mean ± std) of FedAAR with different weighting coefficients *λ* of the prototype guidance regularization loss. The best two results for each metric are highlighted in bold.

λ	Precision (%)	Recall (%)	F1-Score (%)	Accuracy (%)
0.01	74.39 ± 1.07	74.56 ± 2.81	74.08 ± 1.07	88.17 ± 0.61
0.03	75.10 ± 0.82	**74.87** ± **3.39**	**74.53** ± **1.93**	88.55 ± 0.80
0.05	**75.23** ± **1.01**	**75.17** ± **3.92**	**74.70** ± **2.49**	**88.88** ± **1.36**
0.07	74.97 ± 1.08	74.66 ± 4.07	74.18 ± 2.59	88.88 ± 1.65
0.09	**75.94** ± **2.14**	72.50 ± 5.21	72.80 ± 3.85	**88.89** ± **1.67**

## Data Availability

No new data were created nor analysed in this study. Data sharing is not applicable to this article.
